# Effects of age, period, and birth cohort on fall-related mortality in older adults in Brazil from 1980 to 2019

**DOI:** 10.1590/0102-311XEN136524

**Published:** 2025-03-31

**Authors:** José Mário Nunes da Silva, Rita de Cássia de Lima Idalino

**Affiliations:** 1 Departamento de Estatística, Universidade Federal do Piauí, Teresina, Brasil.; 2 Laboratório de Inferência Causal em Epidemiologia, Universidade de São Paulo, São Paulo, Brasil.; 3 Laboratório de Colaboração Estatística, Universidade Federal do Piauí, Teresina, Brasil.

**Keywords:** Falls, Mortality, Age Effect, Period Effect, Cohort Effect, Acidentes por Quedas, Mortalidade, Efeito Idade, Efeito Período, Efeito de Coortes, Accidentes por Caídas, Mortalidad, Efecto Edad, Efecto Periodo, Efecto de Cohortes

## Abstract

Falls in older adults are a major public health problem. This study aimed to estimate the effects of age, period, and birth cohort on fall-related mortality in older adults in Brazil and its geographic regions, by sex, from 1980 to 2019. We conducted an ecological time-series study using data on fall-related deaths in older adults extracted from Brazilian Mortality Information System. Poisson models were adjusted for sex and geographic region to estimate age-period-cohort effects. From 1980 to 2019, Brazil recorded 170,607 fall-related deaths in older adults, with 50.1% occurring in women. More than half of these deaths occurred in the age group of 80 years or older (55%) and in the Southeast Region (52%). We observed an increase in fall-related mortality rates across all age groups and regions, regardless of sex. There was an increased risk of death in all periods after the reference period (2000 to 2004) in all geographic regions and for both sexes. We also observed a gradual increase in mortality risk for men born before 1914 and after 1935 compared to the reference cohort (1930 to 1934). In contrast, we found a protective effect across all birth cohorts for women. There was a consistent increase in fall-related mortality risk among older people in Brazil, posing a public health challenge. The findings highlight the urgent need for implementing public health policies that promotes older adults’ health and prevents fall risks to improve this population’s quality of life.

## Abstract (pt)

As quedas em idosos são um grande problema de saúde pública. Este estudo buscou estimar os efeitos da idade, período e coorte de nascimentos sobre a mortalidade por quedas em idosos no Brasil e suas regiões geográficas, por gênero, no período de 1980 a 2019. Foi realizado um estudo ecológico de séries temporais utilizando dados de óbitos por quedas em idosos extraídos do Sistema de Informações sobre Mortalidade. Os modelos de Poisson foram ajustados para cada gênero e região geográfica para estimar os efeitos idade-período-coorte. De 1980 a 2019, o Brasil registrou 170.607 mortes relacionadas a quedas em idosos, sendo que 50,1% ocorreram em mulheres. Mais da metade desses óbitos ocorreu na faixa etária de 80 anos ou mais (55%) e na Região Sudeste (52%). Observamos um aumento nas taxas de mortalidade por quedas em todas as faixas etárias e regiões, independentemente do gênero. Houve aumento do risco de óbito em todos os períodos após o período de referência (2000 a 2004) em todas as regiões e para ambos os gêneros. Também observamos um aumento gradual no risco de mortalidade para homens nascidos antes de 1914 e depois de 1935 em comparação com a coorte de referência (1930 a 1934). Em contraste, encontramos um efeito protetor em todas as coortes de nascimento para mulheres. Houve um aumento consistente no risco de mortalidade por quedas entre idosos no Brasil, representando um desafio para a saúde pública. Os achados destacam a necessidade urgente de implementação de políticas públicas de saúde que promovam a saúde do idoso e previnam o risco de quedas para melhorar a qualidade de vida dessa população.

## Abstract (es)

Las caídas en las personas mayores son un importante problema de salud pública. Este estudio pretendió estimar los efectos de la edad, el período y la cohorte de nacimientos sobre la mortalidad por caídas en los ancianos en Brasil y sus regiones geográficas, según género, en el período de 1980 a 2019. Se realizó un estudio de series temporales ecológicas con base en datos de muertes por caídas en ancianos extraídos del Sistema de Informaciones de Mortalidad. Los modelos de Poisson se ajustaron por género y región geográfica para estimar los efectos edad-período-cohorte. De 1980 a 2019, Brasil registró 170.607 muertes relacionadas con caídas en ancianos, con un 50,1% en mujeres. Más de la mitad de estas muertes ocurrieron en el grupo etario de más de 80 años (55%) y en la región Sudeste (52%). Se observó un aumento en las tasas de mortalidad por caídas en todos los grupos etarios y regiones, independientemente del género. Hubo un mayor riesgo de muerte en todos los períodos posteriores al período de referencia (2000 a 2004) en todas las regiones y en ambos sexos. También se encontró un aumento gradual en el riesgo de mortalidad para los hombres nacidos antes de 1914 y después de 1935 en comparación con la cohorte de referencia (1930 a 1934). En cambio, se encontró un efecto protector en todas las cohortes de nacimientos de mujeres. Hubo un aumento constante en el riesgo de mortalidad por caídas entre los ancianos en Brasil, lo que representa un desafío para la salud pública. Los hallazgos destacan la necesidad de implementar políticas de salud pública que promuevan la salud de las personas mayores y la prevención del riesgo de caídas para mejorar la calidad de vida de esta población.

## Introduction

Population aging is a global reality that brings significant changes to the epidemiological profile of populations ^1^. In Brazil, increased life expectancy and declining birth rates have led to a rapid growth in the older people proportion ^2^. This natural process causes various physiological and systemic changes in older adults, which can compromise balance and increase the risk of falls ^3^ ^,^ ^4^.

The World Health Organization (WHO) defines a fall as an event which results in a person coming to rest inadvertently on the ground, floor, or other lower level ^5^. Globally, approximately 684,000 individuals die from falls annually ^5^, leading to nearly 17 million years of life lost ^6^. In Brazil, 15.5% of individuals over 60 years fall annually, rising to 22.3% after age 75 ^7^. Those who have fallen before face a 60% to 70% risk of recurrence within a year, with a 20% mortality rate ^8^. Furthermore, healthcare costs for older adults who are victims of falls rise annually ^9^ ^,^ ^10^ ^,^ ^11^.

The literature extensively describes the issue of falls among older adults ^6^ ^,^ ^10^ ^,^ ^12^ ^,^ ^13^. However, few or no studies have explored trends in fall-related mortality rates (FMR), considering the effects of age, period, and birth cohort (APC). The age effect indicates changes in incidence and mortality rates related to chronological age, reflecting the biological and social processes of aging. The period effect considers the influence of historical events and societal changes on the entire population during a specific time, influencing all age groups simultaneously. Meanwhile, the birth cohort effect examines the conditions experienced by a generation born in a specific historical period as they age, enabling the analysis of long-term exposure to risk factors across different cohorts ^14^ ^,^ ^15^.

Traditional studies often restrict their analysis to examining time trends of standardized rates ^10^ ^,^ ^11^ ^,^ ^16^ ^,^ ^17^ ^,^ ^18^ ^,^ ^19^ ^,^ ^20^ ^,^ ^21^, which assess age and period effects but may miss how different generations are affected by evolving health practices and socioeconomic conditions over their lifespans ^14^. APC models enable decomposing these effects, providing valuable insights into the specific influence of each factor, representing an advancement in the trend analyses normally used in epidemiology. Therefore, understanding these dimensions is crucial for resource allocation and the development of more effective and targeted public health interventions aimed at reducing the incidence of falls and their fatal consequences among older people. Thus, we aim to estimate, for the first time, the APC on fall-related mortality among older adults in Brazil and its geographic regions, by sex, from 1980 to 2019.

## Methods

### Study design

An ecological study examining the temporal trends in fall-related mortality among older adults individuals in Brazil and its geographical regions was conducted, encompassing individuals of both sexes aged 60 years or older, spanning from 1980 to 2019.

### Data sources and population

Mortality data were extracted from the Brazilian Mortality Information System (SIM, acronym in Portuguese), coordinated by the Brazilian Health Informatics Department (DATASUS, acronym in Portuguese), available at: http://tabnet.datasus.gov.br/. Mortality information included codes E880 to E888 from the International Classification of Diseases, 9th revision (ICD-9), and codes W00 to W19 from the 10th revision (ICD-10).

Population data were also sourced from DATASUS based on the censuses of 1980, 1991, 2000, and 2010. Population projections for the intercensus years’ July 1st populations were estimated by the Brazilian Institute of Geography and Statistics (IBGE, acronym in Portuguese). The mortality data were adjusted and corrected via the proportional redistribution of records initially classified with unknown age or sex, taking into account each geographical region and study year, before applying the filter for individuals aged 60 years or older in the analysed data ^22^.

### Variables

After obtaining death records and population data, crude and specific annual mortality rates from falls per 100,000 inhabitants were calculated by age group, according to sex and geographic regions, as well as proportional mortality. Subsequently, rates were standardized by age and sex using the direct method, based on the World Standard Population proposed by Segi in 1960, later modified by Doll et al. ^23^.

To improve data stability, ages were assembled into 5-year intervals, starting from 60-64 years and ending with 80 years or older, totaling five age groups. Periods were also grouped into 5-year intervals (1980-1984, 1985-1989, 1990-1994, 1995-1999, 2000-2004, 2005-2009, 2010-2014, and 2015-2019), resulting in eight periods. Finally, birth cohorts ranged from 1900 to 1959, totaling 12 cohorts.

### Statistical analysis

The effects of age, period, and birth cohort were calculated assuming a Poisson distribution for fall-related deaths, with temporal effects (APC) acting multiplicatively on the rate. Equation 1 shows the logarithm of the expected mortality rate (*E[r*_*ij*_ *]*) is a linear function of the effects of age, period, and cohort ^15^ ^,^ ^24^.

$$ln\left(E\left[r_{ij}\right]\right)=ln\left(\frac{\theta_{ij}}{N_{ij}}\right)=\mu +\alpha_{i}+\beta_{j}+\gamma_{k}$$ (1)

In which *θ*_*ij*_ denotes the number of deaths at age *i* and period *j*, *N*_*ij*_ represents the population at risk of death at age *i* and period *j*; *μ* represents the mean effect, *α*_*i*_ the effect of age group *i*, *β*_*j*_ the effect of time period *j*, and *γ*_*k*_ the effect of birth cohort *k*.

The primary challenge in adjusting a model involving age, period, and cohort is their linear relation. For instance, cohort can be inferred when age and period are known. This limitation is known as the “non-identifiability problem”, and there is no consensus on the best solution ^15^. We opted to estimate the parameters of the APC effect via the derivation of estimable functions ^15^ ^,^ ^24^ using Carstensen’s proposed approach ^25^. This involves parameterizing age (a), period (p), and cohort (c) effects in a way that enables their separation, often using techniques such as splines to smoothly model variables while avoiding imposing rigid structures. Thus, models with different forms for age, period, and cohort functions were adjusted: a factor model with one parameter per level, and two models using smoothing functions, natural splines, and B-splines ^25^. Ultimately, the function that provided the best fit was slected.

In this context, the linear trend of the effects consists of two components, the first is the linear effect related to age, and the second, known as the drift, represents the combined linear slope of period and cohort. The longitudinal age trend is the sum of the age and period slopes (*αL + βL*), in which *αL* and *βL* represent the linear trends for age and period, respectively. The term drift reflects the linear trend of the logarithm of age-specific rates, equaling the sum of the period and cohort slopes (*βL + γL*), in which *βL* and *γL* are the linear trends for period and cohort, respectively ^15^ ^,^ ^24^ ^,^ ^25^.

Models were compared using likelihood ratio tests to assess the effects of age, period, and cohort. Sub-models were systematically adjusted to identify the nonlinear effects of these explanatory variables: age, age-drift (cohort drift model), age-cohort, age-period-cohort, and age-drift (period drift model). The best model was obtained based on these comparisons. The adequacy of the final model fit was confirmed using deviance statistics ^25^.

Based on estimates from the final model, period and cohort effects were expressed as relative risks (RR) of fall-related mortality compared to a reference period and cohort, respectively. We used the reference cohort from 1930 to 1935 and the period from 2000 to 2004, chosen because central cohorts and periods typically show greater stability ^26^. The age effects were graphically presented as fall-related mortality rates (per 100,000 inhabitants) by age, adjusted in the cohort, and reference period. We considered values statistically significant via analysis of 95% confidence intervals (95%CI). The APC model estimation analyses were conducted using the *Epi* package in the open-source software R, version 4.3.3 (http://www.r-project.org).

## Results

**Figure 1:**
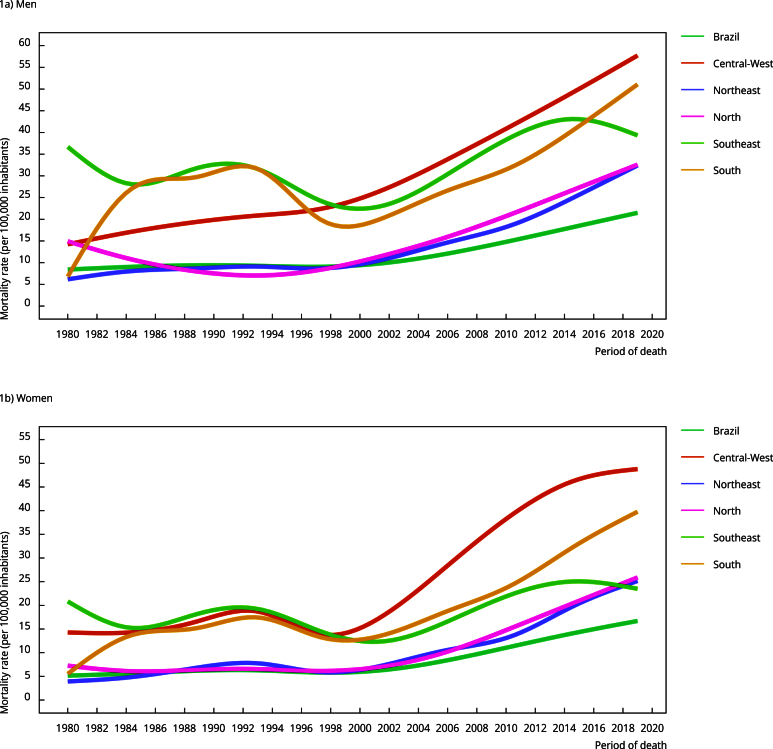
Trend in standardized fall-related mortality rates among older adults, by sex and geographic region, smoothed using third order penalized cubic regression splines, Brazil, 1980-2019.

From 1980 to 2019, Brazil recorded 170,607 fall-related deaths among older adults, with 738 deaths (0.43%) added following proportional redistribution. More than half of these deaths were observed in women (50.1%), in the age group of 80 years or older (55%), and in the Southeast Region (52%). When comparing the years 1980 and 2019, an increase in crude FMR was observed across all age groups and regions, as well as in standardized FMR for both men and women (Table 1). The highest standardized FMR for men (40.8/100,000) and women (28.5/100,000) were observed in 2018 and 2017, respectively. Note that all regions showed a progressive increase in standardized FMR over the study period, with the highest rates evident after 1998 (Figure 1).

**Table 1:** Fall-related mortality rates among older adults (per 100,000 inhabitants), by age, sex, and geographic regions. Brazil, 1980-2019.

Region/Age groups (years)	Men				Women			
	n	%	Rate		n	%	Rate	
			1980	2019			1980	2019
North								
60-64	417	14.0	2.4	14.2	138	5.2	5.1	2.6
65-69	430	14.4	10.5	11.8	195	7.4	8.4	10.5
70-74	422	14.2	8.9	20.6	276	10.1	12.8	15.8
75-79	454	15.2	16.7	44.7	393	14.8	40.5	42.9
≥ 80	1,259	42.2	166.2	156.0	1,656	62.5	10.5	154.3
Standardized rate			22.0	30.4			11.1	24.6
Northeast								
60-64	1,671	11.6	0.3	13.9	813	5.1	1.7	4.7
65-69	1,694	11.8	1.0	18.0	1,028	6.4	1.3	7.7
70-74	1,833	12.7	2.4	26.4	1,555	9.7	5.9	18.3
75-79	2,187	15.2	3.8	43.1	2,242	14.1	4.8	36.7
≥ 80	6,992	48.6	21.9	140.3	10,318	64.7	7.4	140.2
Standardized rate			3.1	31.4			3.2	23.2
Southeast								
60-64	5,814	12.6	2.7	17.1	2,439	5.7	6.4	4.2
65-69	5,873	12.7	7.9	22.9	3,006	7.0	8.4	8.9
70-74	6,387	13.8	23.6	31.5	4,371	10.2	15.2	16.4
75-79	7,492	16.2	59.3	53.2	6,492	15.2	33.7	33.9
≥ 80	20,572	44.6	284.2	161.4	26,336	61.8	99.5	135.9
Standardized rate			38.7	37.7			19.5	22.4
South								
60-64	1,616	10.7	0.0	13.3	598	3.5	1.9	5.3
65-69	1,656	11.0	0.0	22.8	926	5.4	4.9	9.2
70-74	2,009	13.3	3.1	39.8	1,567	9.2	3.8	26.4
75-79	2,502	16.6	8.8	88.3	2,454	14.4	13.3	54.1
≥ 80	7,290	48.4	335	240.0	11,510	67.5	13.5	270.5
Standardized rate			4.4	48.1			5.2	38.7
Central-West								
60-64	702	10.6	1.6	19.0	312	4.4	3.7	6.6
65-69	805	12.2	0.0	25.2	398	5.6	2.3	9.1
70-74	857	12.9	7.0	43.1	606	8.5	11.5	26.5
75-79	975	14.7	24.3	81.4	1,119	15.7	43.4	84.2
≥ 80	3,282	49.6	109.4	287.4	4,677	65.8	67.9	306.2
Standardized rate			14.0	55.1			14.2	45.2
Brazil								
60-64	10,220	12.0	1.5	15.7	4,300	5.0	4.2	4.6
65-69	10,458	12.3	4.2	21.2	5,553	6.5	5.4	8.7
70-74	11,508	13.5	12.2	31.8	8,366	9.8	10.4	19.2
75-79	13,610	16.0	29.6	57.9	12,700	14.9	21.9	41.4
≥ 80	39,395	46.2	147.0	176.0	54,497	63.8	55.0	168.0
Standardized rate			20.0	38.6			11.9	26.6

**Figure 2:**
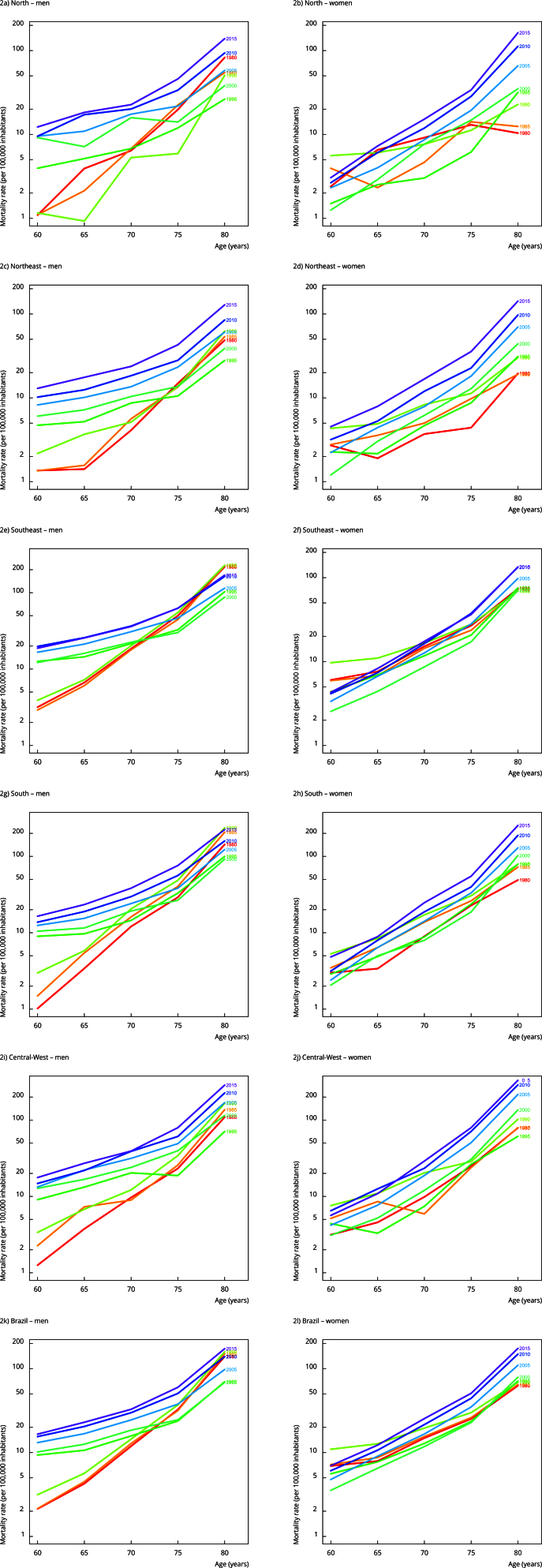
Fall-related mortality rates among older adults by age group, connected within each period, by sex, and geographic region. Brazil, 1980-2019.

**Figure 3:**
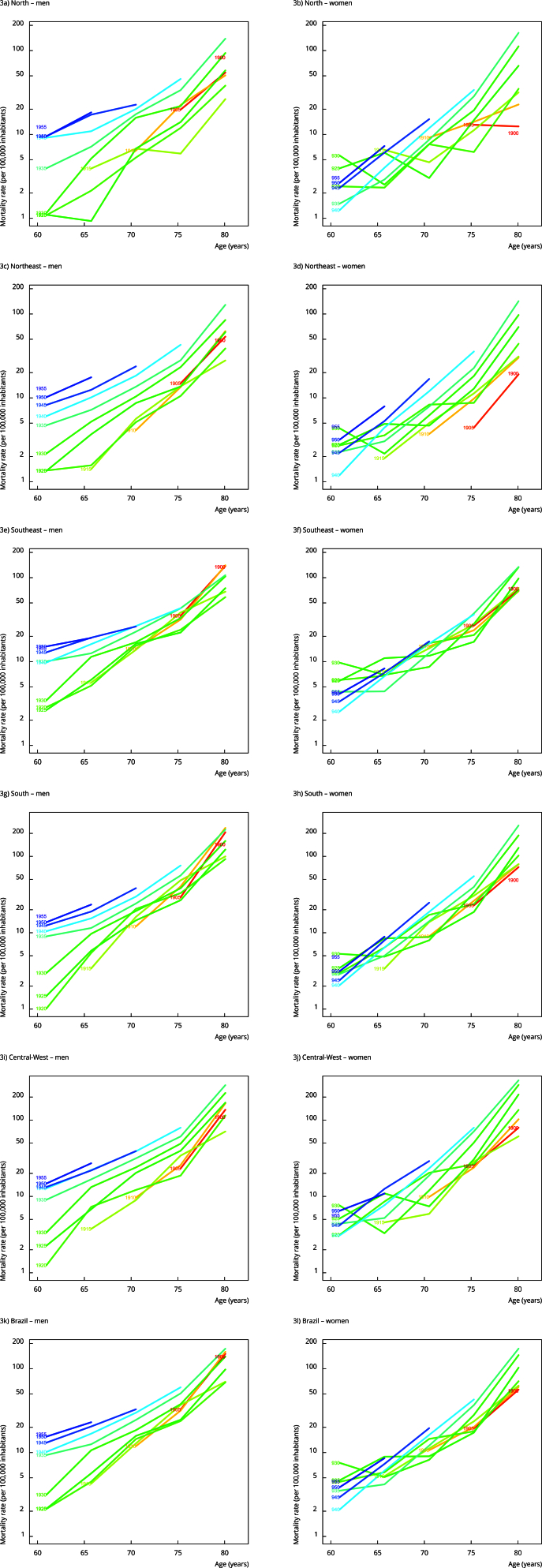
Fall-related mortality rates among older adults by age group, connected within each birth cohort, by sex, and geographic region, Brazil, 1980-2019.

Concerning mortality rates by age group within each period and birth cohort analyzed, there was a progressive increase in FMRs across all age groups for both sexes. This increase was more pronounced from age 75 onwards in Brazil and all geographic regions, regardless of the period (Figure 2). The only exception was observed among women in the North Region, in which a reduction in FMR from 1980 to 1989 starting at age 75 was found (Figure 2b). Additionally, the birth cohorts of 1920, 1930, and 1935 showed higher FMR for both sexes. Notably, men born in the earlier cohorts of 1900 and 1905 had higher FMR in Brazil and all geographic regions (Figure 3).

**Figure 4:**
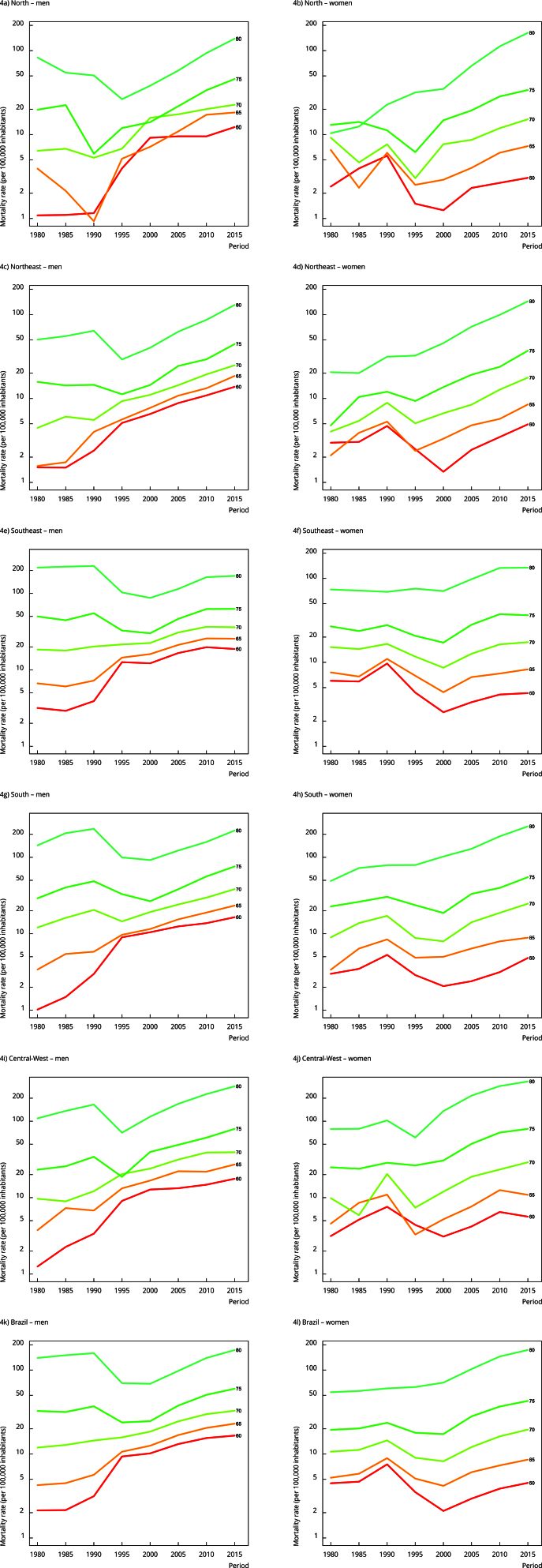
Fall-related mortality rates among older adults per period, connected within each age group, by sex, and geographic regions, Brazil, 1980-2019.

**Figure 5:**
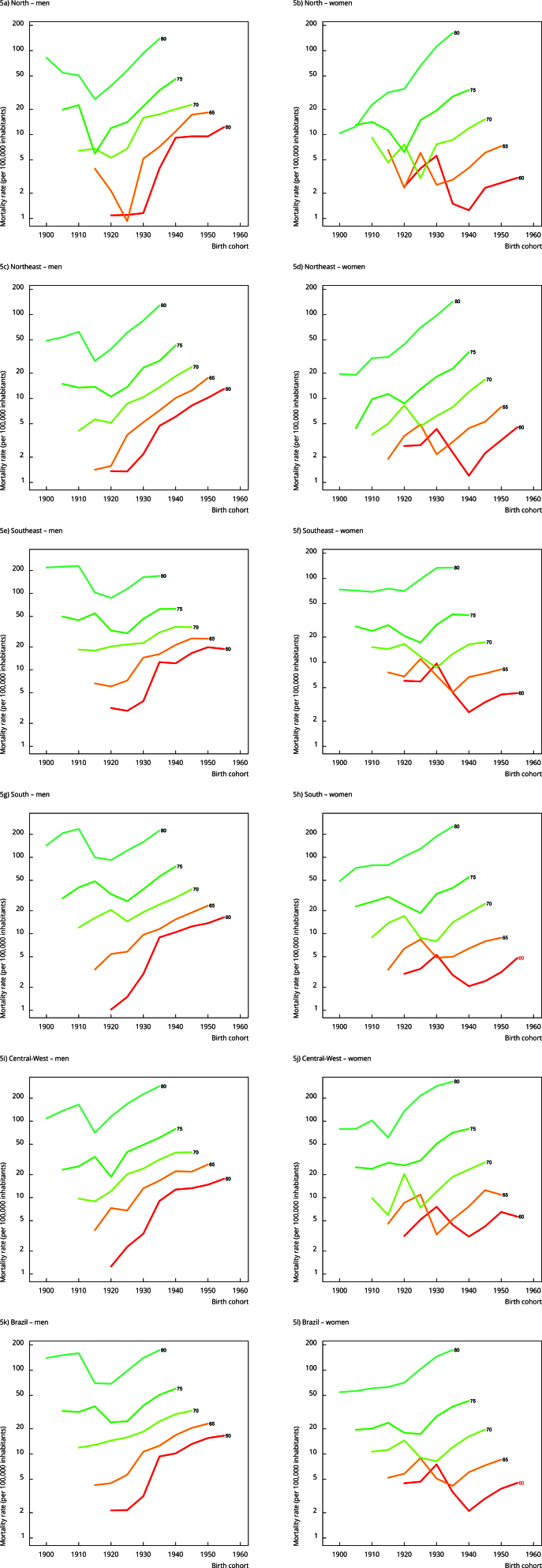
Fall-related mortality rates among older adults by birth cohort, connected within each age group, by sex, and geographic regions. Brazil, 1980-2019.

Regarding mortality, we observed higher FMR across all age groups after the 1995-2000 period, for both sexes, in Brazil and across all geographical regions (Figure 4). For birth cohorts, we found that men belonging to older cohorts experienced a decrease in FMR in the age groups of 75 to 80 years in Brazil and across all geographical regions. For younger birth cohorts, we observed a growing increase in FMR across all age groups in Brazil and all geographical regions, with consistently higher values for male cohorts compared to female cohorts (Figure 5).

Regarding the analyses of age, period, and birth cohort effects for both sexes in Brazil and across all geographical regions, we found that the full APC model best fit the data, with significantly lower deviance (p < 0.0001) (Table 2).

**Table 2:** Model estimates for the age-period-cohort effect on fall-related mortality among older adults, by sex and geographic regions, Brazil, 1980-2019.

Model *	Men					Women				
	Deviance	DF	Deviance **	DF **	p-value ***	Deviance	DF	Deviance **	DF **	p-value ***
North										
Age	827.4	35				927.7	35			
Age-drift ^#^	229.2	34	598.2	1	< 0.001	251.2	34	676.5	1	< 0.001
Age-cohort	62.4	24	166.8	10	< 0.001	165.9	24	85.3	10	< 0.001
Age-period-cohort	44.0	18	18.4	6	< 0.001	28.7	18	137.2	6	< 0.001
Age-period	155.1	28	111.2	10	< 0.001	171.6	28	142.9	10	< 0.001
Age-drift ^##^	229.2	34	74.0	6	< 0.001	251.2	34	79.6	6	< 0.001
Northeast										
Age	3,859.5	35				4.891.0	35			
Age-drift ^#^	850.0	34	3.009	1	< 0.001	654.2	34	4,327	1	< 0.001
Age-cohort	109.3	24	740.7	10	< 0.001	543.0	24	111	10	< 0.001
Age-period-cohort	83.1	18	26.2	6	< 0.001	54.4	18	489	6	< 0.001
Age-period	509.1	28	426.0	10	< 0.001	386.5	28	332	10	< 0.001
Age-drift ^##^	850.0	34	340.8	6	< 0.001	654.2	34	268	6	< 0.001
Southeast										
Age	5,379	35				3,189.6	35			
Age-drift ^#^	4.449	34	930	1	< 0.001	1,939.2	34	1,250	1	< 0.001
Age-cohort	950	24	3,449	10	< 0.001	1,470.9	24	468	10	< 0.001
Age-period-cohort	381	18	569	6	< 0.001	216.1	18	1,255	6	< 0.001
Age-period	3,500	28	3,119	10	< 0.001	915.4	28	699	10	< 0.001
Age-drift ^##^	4,449	34	949	6	< 0.001	1,939.2	34	1,024	6	< 0.001
South										
Age	2,257.2	35				3,051.2	35			
Age-drift ^#^	1,376.3	34	880.9	1	< 0.001	658.8	34	2,392.7	1	< 0.001
Age-cohort	297.1	24	1,079.2	10	< 0.001	508.8	24	149.7	10	< 0.001
Age-period-cohort	140.6	18	156.4	6	< 0.001	63.8	18	445.0	6	< 0.001
Age-period	878.2	28	737.6	10	< 0.001	312.8	28	249.1	10	< 0.001
Age-drift ^##^	1,376.3	34	498.1	6	< 0.001	658.5	34	345.6	6	< 0.001
Central-West										
Age	1,108.1	35				1.382.8	35			
Age-drift ^#^	211.1	34	897.1	1	< 0.001	268.3	34	1,114.5	1	< 0.001
Age-cohort	61.9	24	149.1	10	< 0.001	165.5	24	102.8	10	< 0.001
Age-period-cohort	42.8	18	19.2	6	< 0.001	20.6	18	144.9	6	< 0.001
Age-period	175.0	28	132.2	10	< 0.001	126.2	28	105.6	10	< 0.001
Age-drift ^##^	211.0	34	36.0	6	< 0.001	268.3	34	142.1	6	< 0.001
Brazil										
Age	12,268.1	35				12,132.3	35			
Age-drift ^#^	6,723.0	34	5,545.1	1	< 0.001	3,708.7	34	8,423.6	1	< 0.001
Age-cohort	1,106.8	24	5,616.3	10	< 0.001	2,859.7	24	849.0	10	< 0.001
Age-period-cohort	648.1	18	458.7	6	< 0.001	281.1	18	2,578.6	6	< 0.001
Age-period	4,831.4	28	4,183.3	10	< 0.001	1,823.4	28	1,542.3	10	< 0.001
Age-drift ^##^	6,723.0	34	1,891.6	6	< 0.001	3,708.7	34	1,885.3	6	< 0.001

DF: degree of freedom.

Note: the chosen model is highlighted in bold.

* The models are ordered so that adjacent rows provide tests between the models, culminating in the age-period-cohort model;

** Changes in residual DF and deviance between the models in the current and previous row in the table;

*** p-value of the likelihood ratio test comparing the models in the current and previous row in the table;

^#^ The linear trend of the logarithm of age-specific rates over time equals the sum of the slopes of the period and cohort effects;

^##^ The longitudinal age trend is the sum of the age and period slopes.

**Figure 6:**
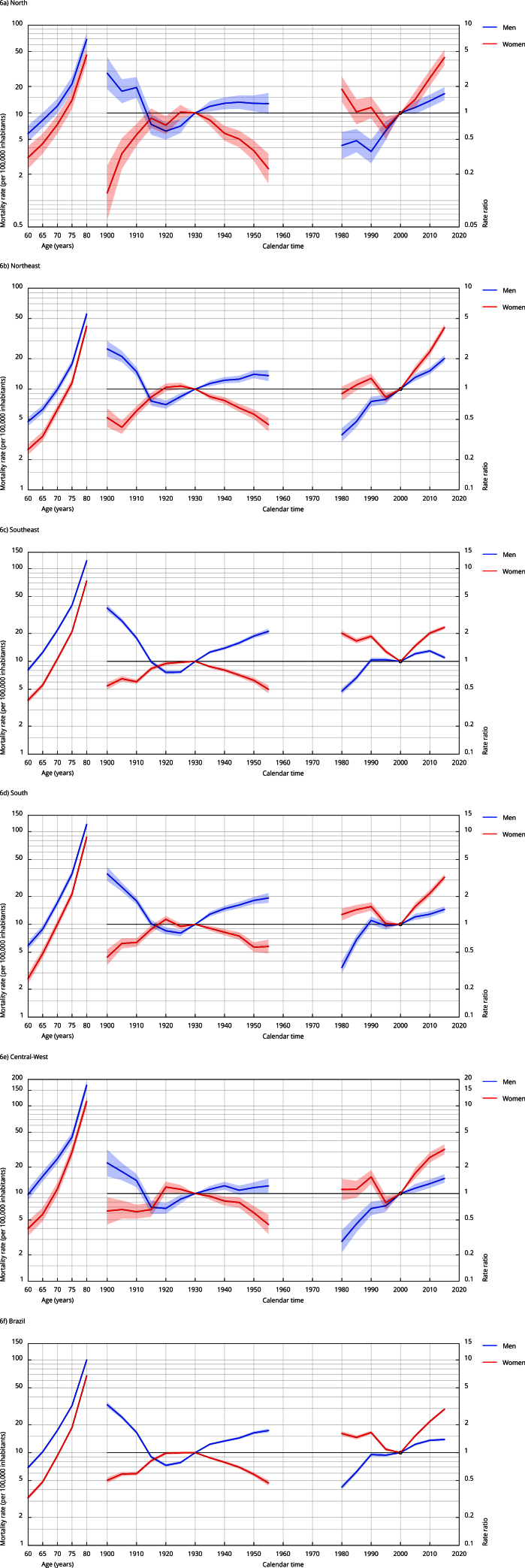
Estimates for age-period-cohort model adjusted for fall-related mortality among older adults, by sex, and geographic regions. Brazil, 1980-2019.

When considering the age effect adjusted for period and birth cohort, we observed a progressive increase in FMR with advancing age across all geographical regions of Brazil, regardless of sex. Notably, the Central-West, South, and Southeast regions showed above-average growth compared to the national average and other geographical regions for both sexes (Figure 6; Tables 3 and 4).

Regarding the period effect adjusted for age and birth cohort, we observed a gradual increase in the risk of death from falls in all periods following the reference period (2000 to 2004) across all geographical regions and for both sexes (Figure 6; Tables 3 and 4).

Regarding birth cohort effect, after adjusting for period and age effects, we observed a gradual increase in the risk of death from falls for men born before 1914 and after 1935, and a decrease in the risk of death from falls for those born from 1915 to 1929, compared to the reference cohort (1930 to 1934), across Brazil and all geographical regions. Overall, for women, we observed a protective effect across all birth cohorts compared to the reference cohort (Figure 6; Tables 3 and 4).

**Table 3:** Estimates of the age-period-cohort model adjusted for fall-related mortality rates among older men by age group, including relative risk for period and birth cohort, by geographic region, Brazil, 1980-2019.

	North	Northeast	Southeast	South	Central-West	Brazil
Age groups (years)	Mortality rate (95%CI)					
60-64	5.8 (4.7-7.19)	4.8 (4.3-5.3)	8.1 (7.7-8.5)	5.9 (5.3-96.5)	9.7 (8.4-11.3)	6.9 (6.6-7.2)
65-69	8.3 (6.8-10.05)	6.3 (5.7-6.9)	12.5 (11.9-13.1)	8.9 (8.1-9.8)	15.7 (13.8-18.0)	10.2 (9.8-10.6)
70-74	12.0 (9.9-14.51)	9.9 (9.0-10.9)	21.7 (20.6-22.8)	17.1 (15.6-18.7)	24.9 (21.8-28.4)	17.32 (16.7-18.0)
75-79	21.4 (17.6-25.97)	17.7 (16.2-19.4)	40.3 (38.4-42.3)	35.0 (32.1-38.3)	43.9 (38.6- 50.0)	32.2 (31.1-33.4)
≥ 80	68.7 (57.6-81.79)	55.4 (51.1-60.0)	121.4 (116.3-126.7)	119.4 (110.4-129.2)	171.4 (152.6-192.4)	99.8 (96.7- 103.1)
Period	Relative risk (95%CI)					
1980-1984	0.42 (0.30-0.61)	0.35 (0.30-0.41)	0.48 (0.44-0.51)	0.34 (0.29-0.40)	0.28 (0.21-0.38)	0.42 (0.40-0.45)
1985-1989	0.48 (0.35-0.66)	0.48 (0.42-0.54)	0.67 (0.63-0.71)	0.68 (0.61-0.77)	0.45 (0.36-0.56)	0.62 (0.59-0.65)
1990-1994	0.36 (0.27-0.50)	0.75 (0.68-0.84)	1.04 (0.98-1.10)	1.10 (1.00-1.22)	0.67 (0.56-0.81)	0.95 (0.91-0.99)
1995-1999	0.63 (0.49-0.80)	0.79 (0.71-0.88)	1.04 (0.99-1.09)	0.97 (0.88-1.06)	0.73 (0.62-0.85)	0.94 (0.90-0.98)
2000-2004	Reference	Reference	Reference	Reference	Reference	Reference
2005-2009	1.15 (0.97-1.37)	1.30 (1.20-1.41)	1.21 (1.16-1.26)	1.21 (1.11-1.31)	1.15 (1.03-1.29)	1.23 (1.19-1.27)
2010-2014	1.37 (1.16-1.62)	1.51 (1.40-1.64)	1.30 (1.24-1.35)	1.29 (1.19-1.39)	1.30 (1.16-1.45)	1.36 (1.32-1.40)
2015-2019	1.66 (1.39-1.97)	2.01 (1.86-2.17)	1.10 (1.05-1.15)	1.45 (1.34-1.56)	1.48 (1.32-1.66)	1.39 (1.34-1.43)
Birth cohort	Relative risk (95%CI)					
1900-1904	2.83 (1.86-4.32)	2.50 (2.09-3.00)	3.76 (3.46-4.08)	3.51 (2.94-4.18)	2.24 (1.57-3.18)	3.29 (3.08-3.52)
1905-1909	1.77 (1.29-2.42)	2.10 (1.85-2.39)	2.73 (2.57-2.90)	2.52 (2.25-2.82)	1.78 (1.42-2.24)	2.42 (2.31-2.53)
1910-1914	1.95 (1.48-2.56)	1.49 (1.34-1.65)	1.78 (1.70-1.88)	1.79 (1.63-1.96)	1.41 (1.18-1.67)	1.65 (1.59-1.72)
1915-1919	0.74 (0.56-0.97)	0.76 (0.68-0.84)	0.99 (0.94-1.04)	1.02 (0.93-1.12)	0.70 (0.59-0.83)	0.90 (0.86-0.93)
1920-1924	0.62 (0.50-0.77)	0.70 (0.64-0.77)	0.76 (0.72-0.80)	0.85 (0.78-0.93)	0.68 (0.59-0.78)	0.73 (0.70-0.76)
1925-1929	0.71 (0.59-0.85)	0.84 (0.78-0.91)	0.77 (0.73-0.80)	0.80 (0.74-0.87)	0.86 (0.77-0.97)	0.78 (0.76-0.81)
1930-1934	Reference	Reference	Reference	Reference	Reference	Reference
1935-1949	1.19 (1.04-1.36)	1.13 (1.06-1.21)	1.26 (1.22-1.30)	1.29 (1.21-1.37)	1.12 (1.02-1.22)	1.23 (1.20-1.26)
1940-1944	1.29 (1.10-1.51)	1.22 (1.14-1.31)	1.39 (1.34-1.44)	1.47 (1.37-1.58)	1.23 (1.10-1.36)	1.33 (1.30-1.37)
1945-1949	1.32 (1.11-1.58)	1.25 (1.16-1.36)	1.58 (1.52-1.65)	1.62 (1.49-1.75)	1.09 (0.97-1.23)	1.44 (1.39-1.49)
1950-1954	1.28 (1.04-1,58)	1.40 (1.28-1.54)	1.87 (1.78-1,97)	1.81 (1.65-1.98)	1.17 (1.01-1.35)	1.64 (1.58-1.70)
1955-1959	1.28 (0.97-1.69)	1.36 (1.20-1.54)	2.11 (1.97-2.26)	1.92 (1.70-2.18)	1.22 (1.01-1.49)	1.73 (1.65-1.82)

95%CI: 95% confidence interval.

**Table 4:** Estimates of the age-period-cohort model adjusted for fall-related mortality rates among older women by age group, including relative risk for period and birth cohort, by geographic region, Brazil, 1980-2019.

	North	Northeast	Southeast	South	Central-West	Brazil
Age groups (years)	Mortality rate (95%CI)					
60-64	3.1 (2.3-4.2)	2.5 (2.2-2.8)	3.8 (3.6-4.1)	2.6 (2.3-3.0)	4.0 (3.3-4.8)	3.3 (3.1-3.4)
65-69	4.5 (3.4-5.8)	3.4 (3.0-3.8)	5.5 (5.2-5.9)	4.8 (4.3-5.4)	5.8 (4.8-6.9)	4.8 (4.6-5.1)
70-74	7.4 (5.8-9.4)	6.3 (5.6-6.9)	10.6 (10.0-11.2)	10.0 (9.0-11.1)	11.3 (9.5-13.3)	9.3 (8.9-9.7)
75-79	14.0 (11.1-17.6)	11.5 (10.5-12.7)	20.9 (19.8-22.1)	21.2 (19.3-23.3)	29.8 (25.6-34.6)	18.7 (17.9-19.4)
≥ 80	45.6 (36.9-56.3)	41.7 (38.3-45.4)	73.6 (70.1-77.2)	86.8 (79.8-94.4)	112.0 (97.9-128.2)	67.4 (65.1-69.9)
Period	Relative risk (95%CI)					
1980-1984	1.86 (1.33-2.60)	0.90 (0.77-1.06)	2.01 (1.87-2.16)	1.28 (1.10-1.48)	1.11 (0.84-1.47)	1.61 (1.52-1.70)
1985-1989	1.03 (0.74-1.43)	1.10 (0.97-1.24)	1.66 (1.56-1.77)	1.44 (1.28-1.62)	1.12 (0.89-1.40)	1.46 (1.39-1.53)
1990-1994	1.15 (0.87-1.53)	1.28 (1.15-1.42)	1.86 (1.76-1.97)	1.56 (1.40-1.73)	1.55 (1.29-1.85)	1.65 (1.58-1.72)
1995-1999	0.68 (0.50-0.91)	0.83 (0.74-0.92)	1.28 (1.21-1.35)	1.02 (0.92-1.13)	0.79 (0.65-0.95)	1.09 (1.04-1.13)
2000-2004	Reference	Reference	Reference	Reference	Reference	Reference
2005-2009	1.42 (1.14-1.77)	1.56 (1.43-1.70)	1.47 (1.40-1.54)	1.56 (1.42-1.70)	1.70 (1.48-1.95)	1.51 (1.46-1.57)
2010-2014	2.50 (2.03-3.07)	2.35 (2.16-2.55)	2.02 (1.92-2.11)	2.16 (1.99-2.35)	2.58 (2.26-2.95)	2.17 (2.09-2.24)
2015-2019	4.27 (3.47-5.27)	4.06 (3.74-4.40)	2.32 (2.22-2.44)	3.22 (2.97-3.49)	3.19 (2.79-3.64)	2.94 (2.84-3.04)
Birth cohort	Relative risk (95%CI)					
1900-1904	0.12 (0.06-0.25)	0.52 (0.42-0.65)	0.54 (0.50-0.59)	0.44 (0.36-0.54)	0.63 (0.44-0.90)	0.50 (0.47-0.54)
1905-1909	0.34 (0.23-0.52)	0.42 (0.36-0.49)	0.65 (0.61-0.69)	0.62 (0.55-0.71)	0.66 (0.52-0.84)	0.59 (0.56-0.62)
1910-1914	0.56 (0.42-0.75)	0.61 (0.54-0.68)	0.60 (0.57-0.64)	0.64 (0.57-0.71)	0.62 (0.52-0.75)	0.59 (0.57-0.62)
1915-1919	0.87 (0.67-1.13)	0.83 (0.75-0.92)	0.84 (0.79-0.88)	0.89 (0.81-0.98)	0.66 (0.54-0.79)	0.82 (0.79-0.85)
1920-1924	0.72 (0.57-0.92)	1.04 (0.95-1.14)	0.95 (0.90-1.00)	1.13 (1.04-1.23)	1.19 (1.03-1.37)	0.99 (0.95-1.02)
1925-1929	1.02 (0.85-1.23)	1.07 (0.99-1.16)	0.98 (0.94-1.02)	0.95 (0.88-1.03)	1.12 (1.00-1.25)	1.00 (0.97-1.03)
1930-1934	Reference	Reference	Reference	Reference	Reference	Reference
1935-1949	0.83 (0.72-0.96)	0.84 (0.79-0.90)	0.88 (0.85-0.91)	0.91 (0.85-0.97)	0.93 (0.85-1.02)	0.88 (0.86-0.90)
1940-1944	0.58 (0.48-0.71)	0.77 (0.71-0.83)	0.81 (0.77-0.84)	0.82 (0.76-0.89)	0.82 (0.73-0.93)	0.79 (0.76-0.81)
1945-1949	0.50 (0.40-0.63)	0.65 (0.59-0.71)	0.71 (0.67-0.75)	0.75 (0.68-0.82)	0.79 (0.69-0.91)	0.70 (0.67-0.72)
1950-1954	0.37 (0.28-0.48)	0.56 (0.50-0.63)	0.62 (0.58-0.67)	0.57 (0.50-0.64)	0.60 (0.51-0.72)	0.58 (0.56-0.61)
1955-1959	0.23 (0.16-0.34)	0.44 (0.38-0.52)	0.50 (0.45-0.55)	0.58 (0.48-0.69)	0.44 (0.34-0.57)	0.47 (0.44-0.51)

95%CI: 95% confidence interval.

## Discussion

As far as we know, this was the first study to analyze the APC effect on fall-related mortality among older adults in Brazil, covering deaths over a 40-year period. Our results highlighted age as the most significant and prominent factor for both sexes across all models analyzed. They also revealed higher fall-related mortality rate in older men, particularly in the Central-West, South, and Southeast regions of the country. The period effect emerged as robust and consistent, showing a significant increase after the 2000-2004 period. Particularly among women, we found inverted V-shaped curves in all geographic regions of Brazil. There were elevated mortality risks among women in the North and Northeast regions from 2015-2019. Additionally, the cohort effect was significant, with a progressive increase in mortality risk among younger cohorts of men born after 1935, returning to levels similar to those before 1915, particularly in the Southeast and South regions, and across Brazil. For women, there was a general reduction in risk across birth cohorts, except for a slight increase in the South and Central-West regions for the 1920-1924 cohorts.

The findings of this study, which show an increase in fall-related mortality among older adults, are consistent with other studies in the literature ^6^ ^,^ ^10^ ^,^ ^16^ ^,^ ^17^ ^,^ ^20^ ^,^ ^27^ ^,^ ^28^ ^,^ ^29^ ^,^ ^30^. This trend can be explained by several interrelated factors. Among these, the increased incidence of falls in this population is notable ^31^, influenced by the decline of the musculoskeletal and sensory systems common with aging ^32^, as well as sedentary lifestyles, which exacerbate this scenario ^3^. The growing prevalence of senile frailty also plays a significant role ^19^, linking advanced age and comorbidities to higher fall-related mortality ^13^ ^,^ ^33^. Furthermore, improvements in the quality of mortality records may have contributed to this finding ^17^.

We must emphasize that aging itself is not a direct cause of falls, although the physiological changes associated with it can increase vulnerability ^16^. Among older adults, falls usually result from a complex interaction between pre-existing medical conditions and unfavorable environments, which may include physical obstacles or a lack of safety adaptations ^3^. These combined factors heighten the risk of falls, which can significantly contribute to the increase in fall-related deaths due to resulting injuries ^16^ ^,^ ^20^ ^,^ ^33^. This underscores the critical importance of preventive strategies that address both the health conditions and the physical environment of older adults ^8^.

In our study, fall-related mortality rate showed an increasing trend in the 75-79 age group and rose sharply in those aged 80 or older, highlighting the need for special attention to the health of older individuals. These numbers will rise in the future with the progressive aging of the population ^20^. Moreover, the increased frailty and severity of falls in individuals aged 75 and above may explain this trend ^19^. Consequently, it would be advisable to implement public health programs both in the community and healthcare settings that prioritize frailty prevention and/or mitigation, especially in those aged 75 or older. Randomized trials and systematic reviews have consistently shown that interventions incorporating resistance or strength training and balance exercises for older adults can reduce the risk of both fatal and non-fatal falls ^34^, as well as the fear of falling ^35^.

Although the literature suggests women have a higher risk of falls ^3^ ^,^ ^36^ due to factors like menopause-related decreases in estrogen and vitamin D levels ^31^ ^,^ ^37^, our study did not necessarily reflect this in higher mortality for women compared to men according to age effects. Similar results have been observed in other studies ^16^ ^,^ ^19^ ^,^ ^20^ ^,^ ^38^ ^,^ ^39^. Notably, across all geographic regions, FMRs were consistently higher among men compared to women, likely due to the circumstances surrounding the falls ^36^.

Several authors suggest higher FMR in men may be due to a higher incidence of outdoor falls, faster leg muscle deterioration, and engagement in more intense activities leading to severe injuries ^16^ ^,^ ^20^ ^,^ ^40^. In addition, men have more comorbidities and worse health compared to women, exacerbating the consequences of fractures ^13^ ^,^ ^19^. Regarding this issue, it is crucial to overcome the cultural belief that illness is a sign of weakness ^16^.

Existing literature indicates an increasing trend in fall-related deaths in both high-income and low-to-middle-income countries, for both sexes, similar to our findings. A recent study conducted in the United States, using trend analysis, found that from 1999 to 2020, there was an annual increase of 3.94% in FMR among adults aged 65 or older ^21^. In Spain, from 2000 to 2015, there was an annual increase in fall-related mortality trends among adults aged over 65 years of 2.5%, with the highest rate found in those aged 75-84 years (6.4%) ^19^. In Canada, from 2001 to 2007, falls accounted for 26% of all unintentional injury deaths, being the leading cause of death in women and the second in men ^41^. High FMR in adults aged 70 and older was also noted across various European countries from 1990 to 2017 ^42^. In China, recent studies have shown a trend of increasing FMR among individuals aged 60 years or older ^20^ ^,^ ^29^ ^,^ ^30^. Similar results have also been observed in trend analyses from other Brazilian studies across different periods and geographic regions analyzed ^10^ ^,^ ^11^ ^,^ ^16^ ^,^ ^17^ ^,^ ^18^ ^,^ ^27^ ^,^ ^28^ ^,^ ^38^.

Our study found varying mortality risks from falls across birth cohorts and sexes. The decrease in FMR up to 1930 for both sexes may be linked to improvements in health patterns and working conditions during those decades. Advancements in healthcare, including new treatments, medications, and vaccination campaigns, contributed to increased life expectancy and better public health ^43^. Thus, the protective effect observed in birth cohorts up to 1930 reflects the benefits of these historical improvements in Brazilian public health.

Furthermore, we must consider the public policies implemented in Brazil after 1935, such as the Consolidation of Labor Laws and the establishment of the Brazilian Unified National Health System (SUS, acronym in Portuguese) ^44^. Specific policies for older people, such as the Active Aging Policy, the National Health Policy for the Elderly, and Statute of the Elderly, had a significant impact on the functioning of this population ^4^ ^,^ ^45^. Such improvements enabled, particularly for women, a reduction in the risk of death in younger cohorts. In contrast, the increased risk of death among men in younger cohorts can be attributed to factors such as nutritional transitions, reduced physical activity, exposure to more hazardous working conditions, and the rise in chronic diseases predisposing to falls ^20^. The results are consistent with previous studies ^29^ ^,^ ^30^.

We also observed geographic disparities in FMR among older adults in Brazil. Sociodemographic factors may contribute to higher FMR in economically developed regions, as the Southeast and South, where urbanization is more prevalent and potentially increases urban obstacles that raise the risk of falls ^19^. The North and Northeast regions have younger age structures compared to other regions, potentially resulting in lower incidence of fatal falls among older people ^3^ ^,^ ^13^. Additionally, the cooler climate in the Southeast and South may lead to less physical activity among older adults, increasing muscular weakness and the likelihood of severe falls that lead to mortality ^20^ ^,^ ^41^.

This study has several limitations. Firstly, being an ecological study, the associations observed at the population level may not reflect individual-level outcomes. Secondly, we relied on secondary data for our analyses, so the quality and completeness of mortality information may vary across the study period and between different regions. This variability could impact the fall-related mortality rate calculations, especially given that we selected data from the transition period between ICD-9 and ICD-10 in Brazil. Finally, analyzing the APC effect is complex and subject to specific assumptions. Therefore, future studies could benefit from including additional variables and employing more sophisticated analysis methods to further explore the results.

Despite these limitations, this study represents the first attempt using an APC model to assess the trend of fall-related mortality over the past four decades in Brazil, disaggregating data by geographic region and sex. This approach is particularly significant as most studies only consider the effects of age and period of death, neglecting the impact of birth cohorts on fall mortality trends. Consequently, we observed an increased risk of fall-related deaths among older adults attributed to the effects of age and period in both sexes and across all Brazilian regions. Regarding the effect of birth cohort, we noted an increased risk only in younger cohorts of men across all geographic regions.

## Conclusion

Given these findings, there is an urgent need to strengthen accident and fall prevention strategies for older people in Brazil. These efforts encompass education, professional training, the creation of safer environments, and interventions tailored to the specific characteristics of each region. Such measures not only aim to curb the rising trend of fall-related deaths among older adults but also have the potential to significantly reduce the economic and social impact of these events in the future, alleviating the burden on society.
